# New paradigm for auditory paired pulse suppression

**DOI:** 10.1371/journal.pone.0177747

**Published:** 2017-05-18

**Authors:** Nobuyuki Takeuchi, Shunsuke Sugiyama, Koji Inui, Kousuke Kanemoto, Makoto Nishihara

**Affiliations:** 1 Neuropsychiatric Department, Aichi Medical University, Nagakute, Japan; 2 Department of Psychiatry and Psychotherapy, Gifu University, Gifu, Japan; 3 Institute of Human Developmental Research, Aichi Human Service Center, Kasugai, Japan; 4 Department of Integrative Physiology, National Institute for Physiological Sciences, Okazaki, Japan; 5 Multidisciplinary Pain Center, Aichi Medical University, Nagakute, Japan; Chiba Daigaku, JAPAN

## Abstract

Sensory gating is a mechanism of sensory processing used to prevent an overflow of irrelevant information, with some indexes, such as prepulse inhibition (PPI) and P50 suppression, often utilized for its evaluation. In addition, those are clinically important for diseases such as schizophrenia. In the present study, we investigated long-latency paired-pulse suppression of change-related cortical responses using magnetoencephalography. The test change-related response was evoked by an abrupt increase in sound pressure by 15 dB in a continuous sound composed of a train of 25-ms pure tones at 65 dB. By inserting a leading change stimulus (prepulse), we observed suppression of the test response. In Experiment 1, we examined the effects of conditioning-test intervals (CTI) using a 25-ms pure tone at 80 dB as both the test and prepulse. Our results showed clear suppression of the test response peaking at a CTI of 600 ms, while maximum inhibition was approximately 30%. In Experiment 2, the effects of sound pressure on prepulse were examined by inserting prepulses 600 ms prior to the test stimulus. We found that a paired-pulse suppression greater than 25% was obtained by prepulses larger than 77 dB, i.e., 12 dB louder than the background, suggesting that long latency suppression requires a relatively strong prepulse to obtain adequate suppression, different than short-latency paired-pulse suppression reported in previous studies. In Experiment 3, we confirmed similar levels of suppression using electroencephalography. These results suggested that two identical change stimuli spaced by 600 ms were appropriate for observing the long-latency inhibition. The present method requires only a short inspection time and is non-invasive.

## Introduction

When a sensory stimulus is repeatedly presented, responses to it become weak over time, which is generally considered to be caused by inhibitory processes that prevent an overflow of irrelevant information, sometimes referred to as sensory gating. Several methods are utilized to observe such changes in brain responsiveness in humans, including auditory P50 suppression and prepulse inhibition (PPI).

Such measurements are important clinically, because several previous studies have shown that patients with schizophrenia have deficits in P50 suppression [1–6]. For example, it was reported that positive symptoms of schizophrenia, such as auditory hallucinations [7, 8], and also the risk for developing schizophrenia [9] have a relationship to impairment of P50 suppression. Other clinical conditions also known to be related to impairment of sensory suppression include bipolar disorder [10], panic disorder [11], epilepsy [12], and attention-deficit/hyper-active disorder [13]. Hence, P50 suppression deficit has been speculated to be associated with varied cognitive impairments in a wide range of disorders and their symptoms.

P50 suppression is an electrophysiological measure of cortical responses to two consecutive identical auditory stimuli, in which amplitudes of positivity around 50 ms (P50) are compared between the first and second stimuli. For one series, a pair of clicks is usually presented with an inter-pair interval ranging from 8 to 12 seconds, with an average of 10 seconds. The first stimulus is termed the conditioning stimulus, or S1, and the second following S1 given 500 ms later is termed the testing stimulus, or S2. Stimuli consisting of 100–200 paired clicks are presented, and the degree of suppression is evaluated by P50 amplitudes elicited by S1 and S2 as values presented as either ratios or differences [4, 14].

Change-related cortical responses are specifically elicited by abrupt changes in a continuous sensory stimulus and can be recorded very clearly with magnetoencephalography (MEG) or electroencephalography (EEG) [15–18]. Because these activities show a very high test-retest reliability with an r value of approximately 0.9 [19–21], the change-related cortical response is considered to be a reliable measure of higher order brain functions. Recently, we developed a method to observe sensory suppression that utilizes change-related cortical responses [19, 22, 23]. When a change stimulus (test stimulus) is preceded by a weak change stimulus (prepulse), the test response is clearly suppressed in a manner similar to the PPI of startle reflexes [19, 22]. Therefore, we refer to the phenomenon as PPI of auditory evoked cortical responses. Since suppression of the test response occurs with a very weak or no cortical response by the prepulse, it appears to represent an active inhibitory process [23].

When the interval between the prepulse and test stimulus is manipulated, the degree of suppression shows several peaks at different latencies, suggesting the existence of multiple mechanisms of suppression. In the present study, we focused on long-latency suppression peaking at an interval around 500–700 ms. Given that such a long-latency mechanism may be involved in regulation of the firing of pyramidal cells, which particularly prevent their runaway [24], this may be an important clinical test for diseases with abnormal neuronal firing such as epilepsy. However, in a previous study, the threshold of long-latency suppression of auditory change-related responses was so high that the degree of suppression was approximately 10% on average in healthy volunteers when a weak change stimulus was used as a prepulse [23]. Such a value does not appear to be suitable for clinical situations. Another problem of measuring long-latency suppression is that it takes more time to record as compared to short-latency suppression. Therefore, in the present study, we conducted three different experiments to establish a new paradigm for long-latency paired pulse suppression for use in clinical applications.

## Methods

This study was approved in advance by the Ethics Committee of the National Institute for Physiological Sciences, Okazaki, Japan, and all subjects provided written consent prior to participation. None had a history of mental or neurological disorders, or substance abuse in the most recent 5 years, and all were free of medication at the time of testing.

### Auditory stimuli

**Fig 1:**
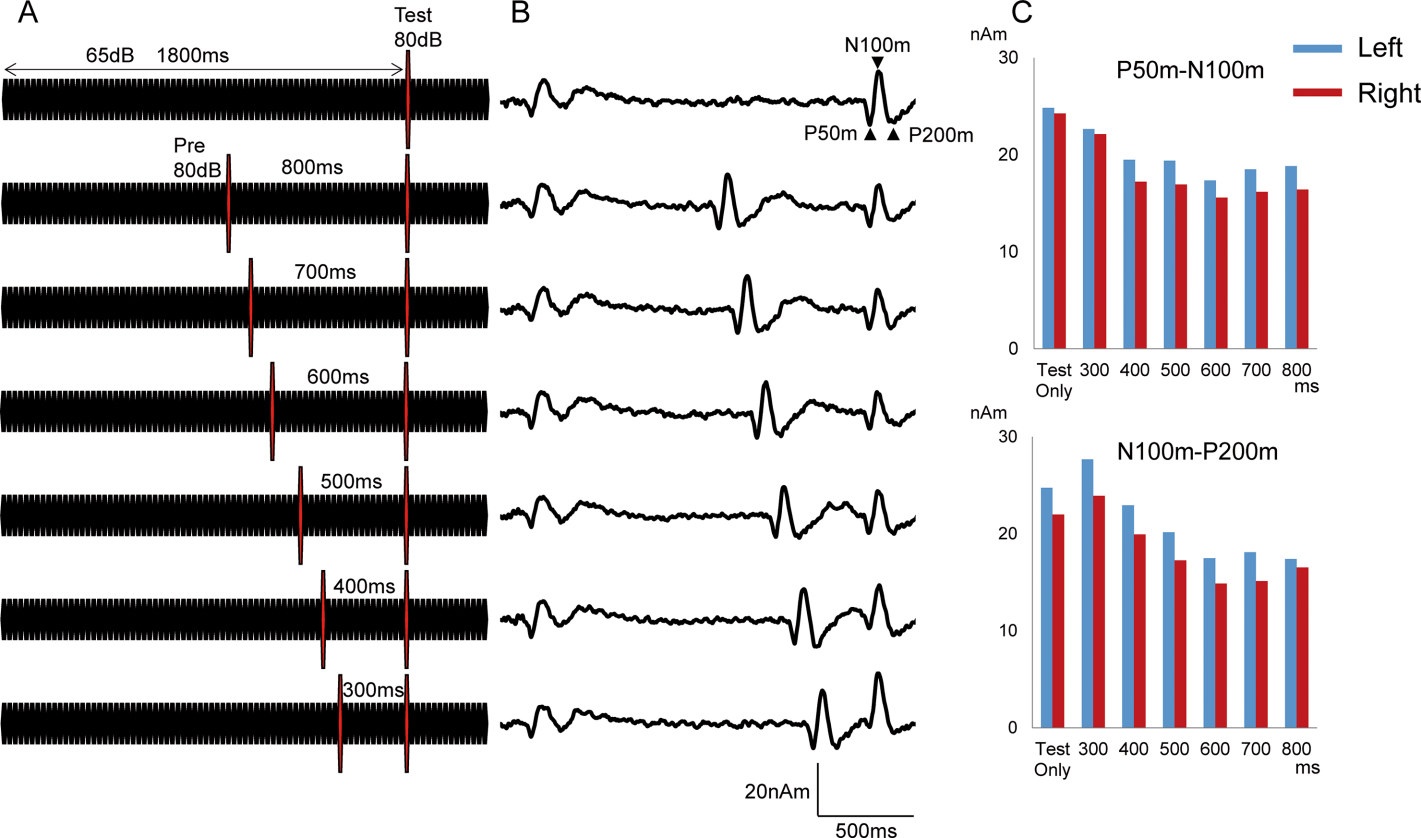
Paired stimulation paradigm using auditory change-related cortical responses. A. The sound stimuli consisted of 86 repeats of a 25-ms pure tone at 65 dB SPL. For the Test stimulus, a 25-ms sound at 80 dB was inserted at 1800 ms. The conditioning stimulus was also a 25-ms pure tone at 80 dB presented at 300–800 ms before the Test stimulus. B. Grand-averaged waveforms for the subjects. C. Mean P50m-N100m and N100m-P200m amplitudes for each condition.

Repeats of a 25-ms pure tone at a frequency of 800 Hz were used, as described elsewhere [17]. The control sound (Control) was 86 repeats of the 25-ms tone at 65 dB SPL of sound pressure, yielding 2150 ms in total duration (Fig 1). For the test sound (Test), a 25-ms tone of 80 dB was inserted at 1800 ms. The conditioning stimulus (Prepulse) was also 25 ms in duration and presented before the test stimulus. We referred to the time between the conditioning and test stimuli as the conditioning-test interval (CTI). Sound stimuli were presented binaurally through ear pieces (E-A-Rtone 3A, Aero Company, Indianapolis, IN).

### Recordings

Each subject sat in a chair and watched a silent movie on a screen 2 m in front of them, and was instructed to ignore sound stimuli throughout the experiment. Magnetic signals were recorded using a 306-channel whole-head type MEG system (Vector-view, ELEKTA Neuromag, Helsinki, Finland), which was comprised of 102 identical triple sensor elements. Each sensor element consisted of 2 orthogonal planar gradiometers and 1 magnetometer coupled to a multi-superconducting quantum interference device (SQUID), and thus provided 3 independent measurements of the magnetic fields. In the present study, we analyzed MEG signals recorded from 204 planar-type gradiometers, which were sufficiently powerful to detect the largest signal just over local cerebral sources. Signals were recorded with a bandpass filter of 0.1–300 Hz and digitized at 1000 Hz. Analysis was conducted from 100 ms before to 2300 ms after the onset of the stimuli. Epochs with MEG signals larger than 2.7 pT/cm were rejected from averaging. The waveform was digitally filtered with a bandpass filter of 1.5–75 Hz and a notch filter at 35–45 Hz.

In Experiment 3, we conducted EEG recordings in a quiet, electrically shielded room. Each subject sat in a comfortable chair and watched a silent movie on a monitor 2 m in front of them, and was instructed to ignore the sound stimuli throughout the experiment. An exploring electrode was placed at Fz and was referred to the linked mastoids (P9-P10) of the 10–10 system. EEG derivation was determined based on the dipole location of the auditory change-related response in the superior temporal gyrus and dipole orientation toward the frontal midline [24]. Impedance for all electrodes was kept under 10 kΩ. EEG artifact rejection was set at 150 μV. Signals were filtered with a band pass filter of 0.5-50Hz. The sampling rate was 1000 Hz. For each condition, over 200 artifact-free epochs were averaged for each stimulus.

### Procedures

#### Experiment 1

The effects of the CTI on paired pulse suppression were examined using 11 healthy volunteers (7 males, 4 females; 20–53 years old, 36.0 ± 11.1 years). We manipulated the CTI from 300 to 800 ms to select the best for exerting suppression. The Prepulse of 80 dB was presented at 300, 400, 500, 600, 700, or 800 ms before the test stimulus. Therefore, this experiment used 7 different sound patterns, the Test alone and the Test + Prepulse at 1000, 1100, 1200, 1300, 1400, and 1500 ms (Fig 1), which were randomly presented. At least 100 artifact-free epochs were averaged for each sound.

#### Experiment 2

We examined the effects of prepulse sound pressure on the suppression with a CTI of 600 ms using 11 healthy volunteers (8 males, 3 females; 20–53 years old, 34.1 ± 11.0 years). The sound pressure of the Prepulse was 68, 71, 74, 77, or 80 dB. Therefore, in Experiment 2 the Test response was compared among 6 conditions, the Test alone and 5 combinations of Test + Prepulse, with the 6 sounds randomly presented. At least 100 artifact-free epochs were averaged for each sound.

#### Experiment 3

We examined whether similar suppression could be detected using EEG with 11 subjects (7 males, 4 females; 26–53 years, 36.1 ± 9.7 years). Three different sounds were used, Test alone, and Test + Prepulse at 300 and 600 ms. The Prepulse was 80 dB in SPL.

### Analysis

Dipole analyses were performed for the Test alone response using the Brain Electrical Source Analysis (BESA) software package (NeuroScan, Mclean, VA), as described elsewhere [25, 26]. The equivalent current dipole for the main component of N100m was estimated in each hemisphere. The obtained two-dipole model obtained was applied to MEG signals for all conditions to simplify the data analysis. The peak latency and amplitude were measured using the source strength waveform.

The test stimulus evoked a triphasic response with peaks at approximately 50 (P50), 100 (N100), and 200 (P200) ms, and we measured the peak amplitudes in time windows of 50–80, 80–150, and 150–250 ms, respectively. It is known that the latency of change-related responses is dependent on the magnitude of the change [16]. Therefore, longer latency periods were allowed for the Prepulse-evoked responses in Experiment 2, e.g., 50–110 ms for the P50m latency. Peak-to-peak amplitudes were calculated for P50m-N100m and N100m-P200m, and used for estimating the degree of suppression. This procedure minimizes problems due to a base line shift [27]. The percent inhibition of the test response by a prepulse (%inhibition) was calculated as follows: (Test alone response–(Prepulse + test response) / Test alone response * 100. The amplitude of the test response was compared among the conditions using two-way repeated measures ANOVA with Prepulse and Hemisphere as the independent variables. In order to compare differences between conditions, post-hoc multiple comparisons were done with Bonferroni-adjusted t-tests. All statistical analyses were performed with 0.05 as the level of significance. Data are expressed as the mean ± standard deviation.

## Results

### Experiment 1

**Fig 2:**
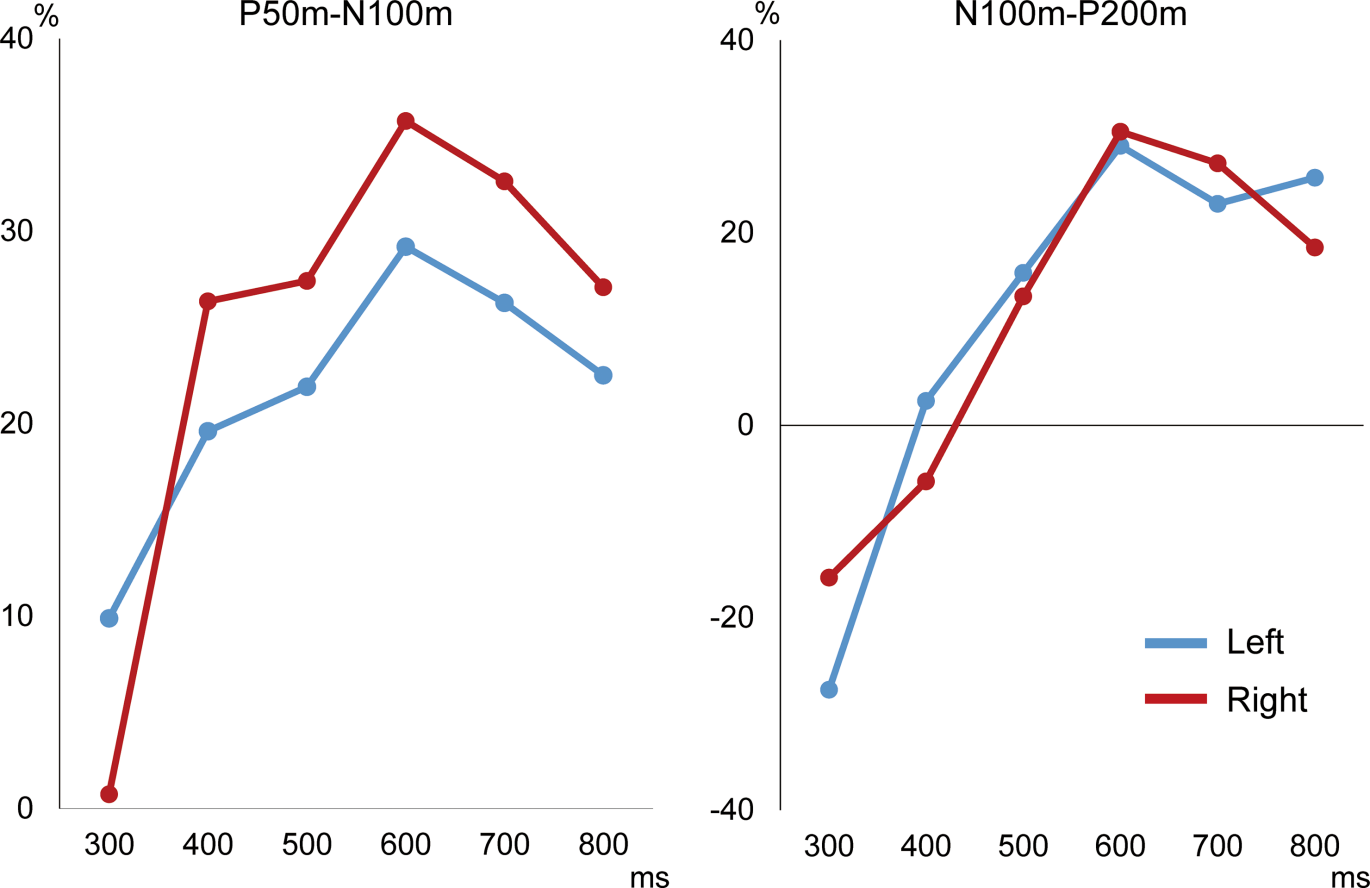
Rate of inhibition against conditioning-test intervals. For both measurements, the inhibition rate peaked at the 600-ms interval.

An abrupt increase in sound pressure elicited clear triphasic responses. The grand-averaged waveforms are shown in Fig 1, while the peak amplitude and %inhibition for each condition are listed in Table 1. The results of two-way ANOVA (Hemisphere X Prepulse) showed that Prepulse was a significant factor for determining the Test P50m-N100m response (F_6,60_ = 10.76, p = 4.30*10^−8^), whereas Hemisphere was not (F_1,10_ = 0.42, p = 0.53). As shown in Fig 2, %inhibition was largest for prepulse at 600 ms prior to the test onset in both hemispheres. Post-hoc tests showed that the Test + Prepulse responses were significantly smaller than the response to the Test alone for CTIs of 500 (p = 0.006), 600 (p = 0.007), 700 (p = 0.008), and 800 (p = 0.027) ms.

**Table 1:** Rates of amplitude and inhibition in Experiment 1.

	P50/N100				N100/P200			
Test	Amplitude		Test/ Test alone PPI%		Amplitude		Test/ Test alone PPI%	
	Left	Right	Left	Right	Left	Right	Left	Right
Control	24.8(11.7)	24.3(10.6)			24.7(12.2)	22.0(8.00)		
300	22.7(12.1)	22.1(7.07)	9.88(24.1)	11.9(31.2)	27.7(15.1)	23.9(9.35)	-27.5(88.4)	-14.8(40.1)
400	19.5(10.3)	17.2(6.32)	19.6(28.5)	16.3(13.1)	22.9(12.3)	19.9(5.75)	2.52(34.1)	3.55(27.1)
500	19.4(9.31)	16.9(7.55)	21.9(15.3)	13.1(16.0)	20.2(10.1)	17.3(5.90)	15.85(24.1)	16.4(28.1)
600	17.3(8.17)	15.6(7.56)	29.2(11.6)	21.7(17.4)	17.5(9.23)	14.9(5.84)	29.0(12.2)	32.2(17.2)
700	18.5(9.27)	16.2(8.52)	26.3(11.9)	23.3(21.7)	18.1(8.29)	15.1(6.17)	23.0(13.5)	30.6(17.3)
800	18.8(8.32)	16.4(6.47)	22.5(17.1)	17.0(24.0)	17.4(8.93)	16.5(4.91)	25.7(25.8)	18.7(26.6)
Pre			Test/Pre PPI%				Test/Pre PPI%	
300	25.5(13.1)	21.6(9.86)	0.738(19.6)	-12.4(31.7)	26.4(13.8)	21.4(6.77)	-8.34(41.6)	-15.8(38.5)
400	24.0(12.4)	21.9(8.76)	26.4(24.6)	17.6(13.1)	26.8(14.9)	20.4(7.44)	7.39(23.1)	-5.85(36.1)
500	22.6(11.0)	20.3(6.94)	27.4(19.6)	16.7(16.0)	24.9(12.8)	20.7(7.07)	18.5(9.47)	13.4(22.1)
600	24.0(14.0)	21.1(7.72)	35.7(20.1)	29.5(17.4)	24.4(12.8)	21.1(6.72)	26.5(22.6)	30.5(17.2)
700	24.6(12.0)	21.4(7.39)	32.6(15.6)	27.4(21.7)	26.1(13.6)	21.2(8.01)	26.5(17.0)	27.2(19.5)
800	24.1(11.7)	21.0(6.88)	27.1(21.9)	22.1(24.0)	24.2(15.0)	20.5(5.13)	17.7(25.2)	18.5(18.5)

As for the N100m-P200m amplitude, the Test-evoked responses were significantly different among the CTI conditions (F_6,60_ = 12.65, p = 3.65*10^−9^), though not between hemispheres (F_1,10_ = 1.13, p = 0.32). When compared to the Test alone condition, post-hoc test results showed that the Test + Prepulse conditions with CTIs of 600 (p = 0.007), 700 (p = 0.009), and 800 (p = 0.020) ms were significantly smaller in amplitude. Similar to the P50m-N100m measure, prepulse with a CTI of 600 ms exerted maximum suppression (Fig 2). The mean amplitude of N100m-P200m is listed in Table 1.

**Fig 3:**
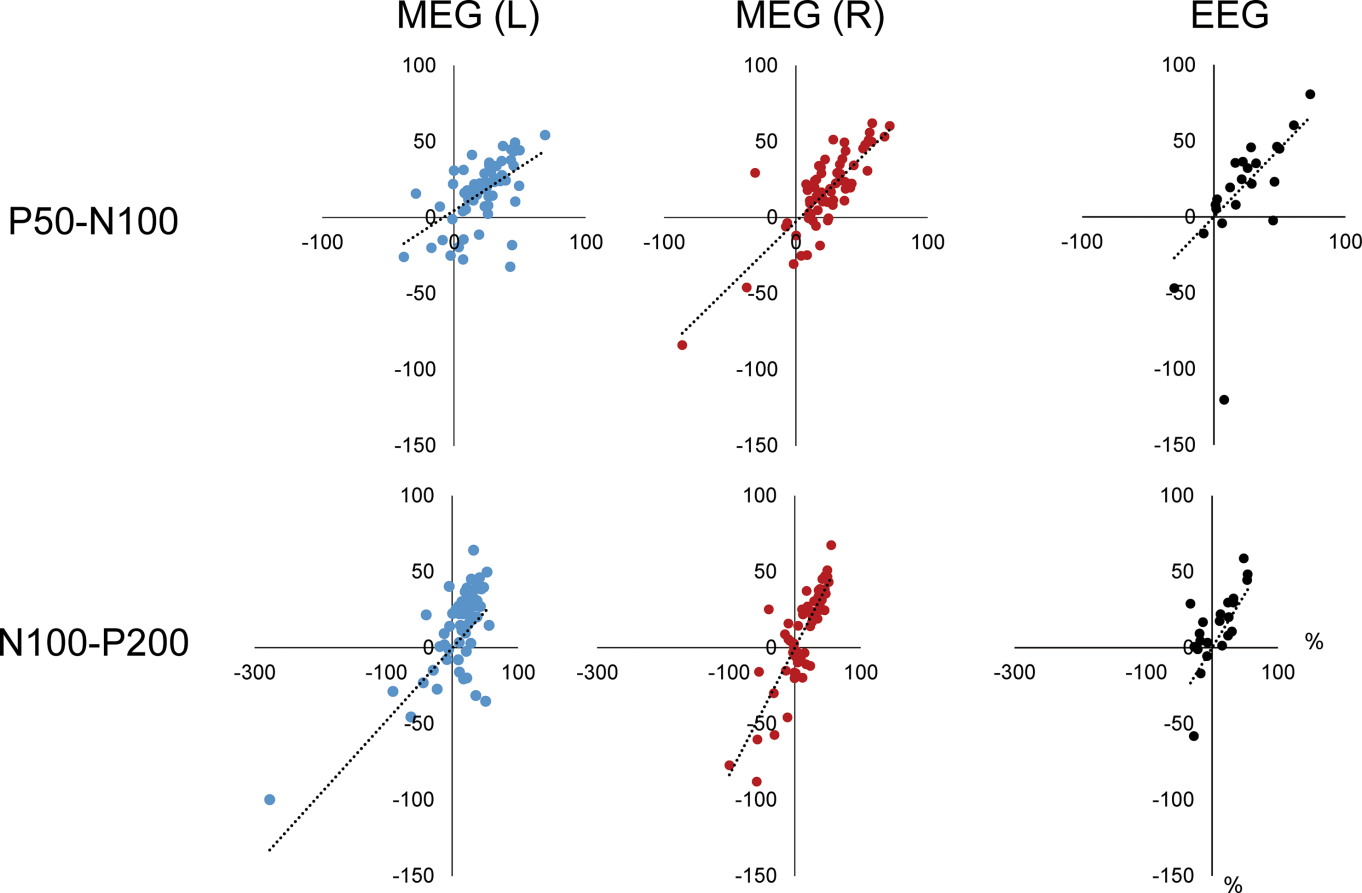
Correlation of inhibition rate between two calculations, Test alone response-based and Conditioning response-based. Plots show the relationship of inhibition rate between the test response / conditioning-evoked response (y axis) and test response / test alone-evoked response (x axis).

The amplitude of the Prepulse-evoked response did not differ significantly between hemispheres (F_1,12_ = 1.17, p = 0.3) or among prepulse conditions (F_5,60_ = 0.81, p = 0.55) for P50m-N100m. Also, neither variable had an effect on the N100m-P200m amplitude (F_1,10_ = 2.11, p = 0.18 for Hemisphere and F_5,50_ = 1.06, p = 0.39 for Prepulse) (Table 1). Since the Prepulse-evoked response did not differ in amplitude among the 6 conditions, it is considered possible to use it as the control response instead of the Test alone response. In order to assess this possibility, the correlations of %inhibition with the Test alone-based and Prepulse-based inhibition values were examined. Fig 3 presents scatter plots of %inhibition for the 2 calculations. There was a significant correlation for both the left (correlation coefficient, r^2^ = 0.30, p < 0.0001) and right (r^2^ = 0.66, p < 0.0001) hemispheres. The results for N100m-P200m were similar, as there was a significant positive correlation for the left (r^2^ = 0.37, p < 0.0001) and right (r^2^ = 0.73, p < 0.0001) hemispheres.

The peak latency was not different between the hemispheres for P50m (F_1,10_ = 0.87, p = 0.37), N100m (F_1,10_ = 0.66, p = 0.43), and P200m (F_1,10_ = 1.59, p = 0.24), or among the Prepulse conditions for P50m (F_6,60_ = 1.44, p = 0.22) and N100m (F_6,60_ = 0.89, p = 0.51). In contrast, the latency of P200m was significantly different among the conditions (F_6,60_ = 2.30 p = 0.046). However, the latency for all Test + Prepulse conditions was not significantly different from the Test alone condition. Mean peak latencies for all conditions are listed in Table 2.

**Table 2:** Latency in Experiment 1.

Test	P50		N100		P200	
	Left	Right	Left	Right	Left	Right
Control	64.3(6.41)	64.6(8.07)	111.2(9.84)	109.4(9.04)	192(29.6)	185(20.0)
300	61.6(7.54)	60.6(9.29)	113.0(11.2)	109.4(9.11)	204(27.0)	194(22.5)
400	59.6(8.07)	65.8(7.44)	112.4(9.94)	112.4(12.2)	203(23.8)	190(21.2)
500	60.1(7.61)	61.6(8.71)	113.5(9.61)	108.7(11.2)	192(30.2)	195(23.8)
600	63.5(7.57)	62.3(7.58)	110.0(10.9)	110.4(9.10)	209(28.2)	192(28.2)
700	62.8(7.56)	67.1(7.38)	109.3(13.2)	108.3(10.3)	184(23.0)	182(15.3)
800	63.3(8.15)	63.0(8.98)	109.8(7.86)	109.0(13.9)	198(32.5)	183(21.3)
Pre						
300	61.6(8.23)	60.3(8.20)	109.8(9.38)	111.0(11.3)	196(30.2)	193(28.2)
400	63.6(7.13)	60.8(6.59)	109.8(10.6)	112.0(11.4)	186(19.5)	188(25.2)
500	62.4(7.00)	62.5(7.55)	110.8(10.6)	109.9(11.6)	191(21.0)	198(23.7)
600	62.3(6.23)	60.5(7.01)	111.4(12.7)	109.5(11.4)	187(25.0)	178(16.6)
700	61.4(4.67)	60.5(6.79)	110.6(12.1)	110.3(10.8)	192(30.4)	195(34.8)
800	62.3(8.15)	61.9(7.03)	109.9(11.0)	111.2(11.1)	194(27.7)	189(20.8)

### Experiment 2

**Fig 4:**
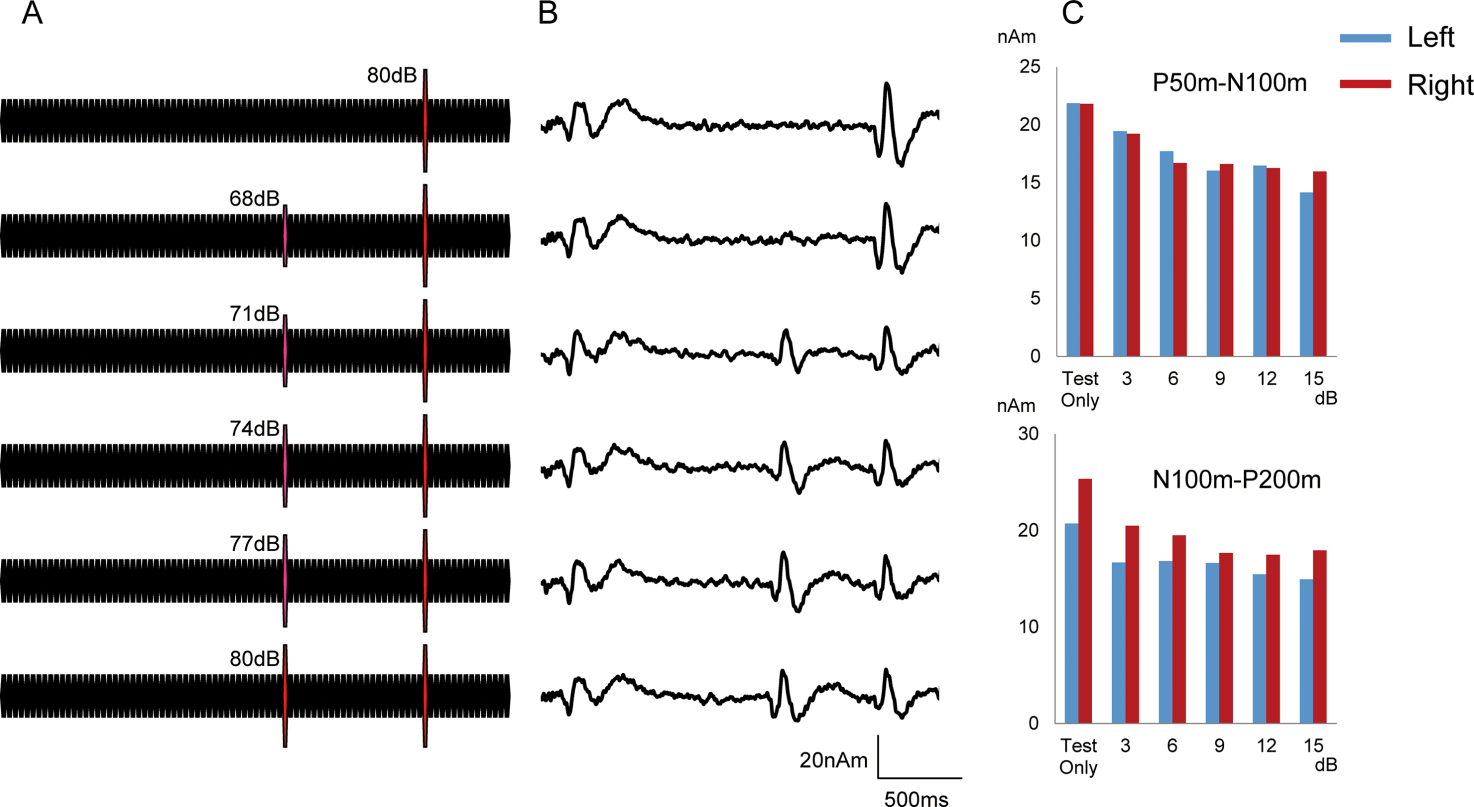
Effects of sound pressure on inhibition. A. Stimulation paradigm used in Experiment 2. The sound stimuli consisted of 86 repeats of the 25-ms tone at 65 dB. The Test sound of 80 dB was inserted at 1800 ms. The conditioning stimulus was 65–80 dB of sound pressure and presented at 600 ms before the test stimulus. B. Grand-averaged waveforms. C. Mean amplitude for each condition.

**Fig 5:**
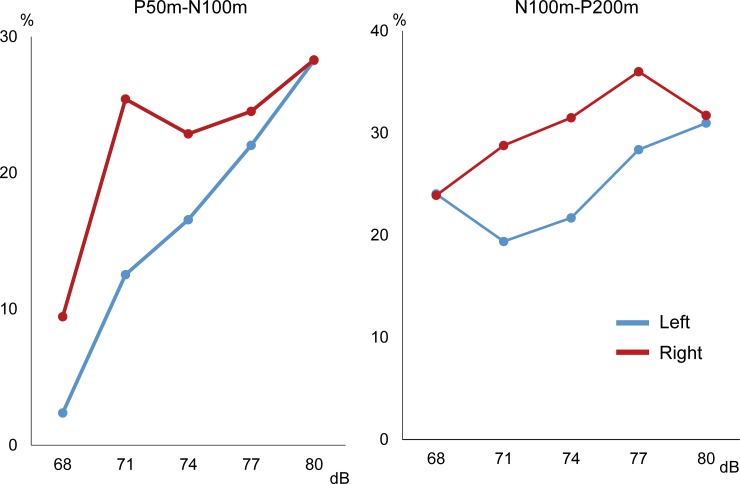
Rate of inhibition against sound pressure level of Prepulse. Inhibition rate results obtained in Experiment 2.

Grand-averaged waveforms are shown in Fig 4. The peak amplitude and %inhibition for each condition are listed in Table 3. The results of two-way ANOVA (Hemisphere X Sound pressure) showed that sound pressure was a significant factor for determining the amplitude of the Test-evoked response for P50m-N100m (F_5,50_ = 11.2, p = 2.83*10^−7^) and for N100m-P200m (F_5,50_ = 13.8, p = 1.79*10^−8^), whereas Hemisphere was not a significant factor for P50m-N100m (F_1,10_ = 0.003, p = 0.96) or N100m-P200m (F_1,10_ = 1.47, p = 0.25). As shown in Fig 5, expect for N100m-P200m in the right, the inhibitory effect was largest for the 80-dB Prepulse in both hemispheres and the 77 dB Prepulse in the right for N100m-P200m (Table 3). As in our previous study [22], stronger prepulses resulted in greater inhibition; e.g., %inhibition greater than 20% was obtained by a 74-dB or stronger Prepulse.

**Table 3:** Rates of amplitude and inhibition in Experiment 2.

	P50/N100				N100/P200			
Test	Amplitude		Test/ Test alone PPI%		Amplitude		Test/ Test alone PPI%	
	Left	Right	Left	Right	Left	Right	Left	Right
Control	27.4(21.3)	24.1(13.0)			25.7(14.7)	27.5(17.7)		
+3dB	24.3(20.6)	22.4(10.9)	8.16(24.2)	0.914(26.5)	20.6(16.1)	22.5(15.9)	26.9(16.2)	24.1(16.4)
+6dB	22.5(16.2)	20.2(10.2)	11.6(21.0)	16.0(16.1)	20.8(14.2)	22.4(16.0)	21.0(10.9)	24.8(19.9)
+9dB	19.6(15.7)	19.0(11.3)	23.0(22.6)	18.8(19.7)	20.3(16.2)	20.0(14.0)	28.2(15.3)	28.0(23.3)
+12dB	20.8(15.9)	18.8(11.6)	20.8(13.8)	19.4(25.8)	19.1(15.4)	20.7(15.3)	30.3(14.9)	30.0(24.1)
+15dB	17.4(14.0)	19.2(12.6)	34.9(11.9)	19.3(26.4)	18.2(14.5)	20.8(15.2)	37.1(19.8)	25.6(18.4)
Pre								
+3dB	8.40(4.67)	7.55(5.75)			9.74(6.06)	9.44(5.91)		
+6dB	14.5(9.35)	14.4(6.87)			16.9(10.5)	16.0(8.33)		
+9dB	17.7(11.5)	18.6(8.41)			21.3(12.6)	23.2(13.7)		
+12dB	20.4(16.7)	22.0(9.33)			22.3(15.2)	25.9(13.9)		
+15dB	22.0(15.1)	24.1(11.9)			22.6(12.6)	26.4(17.2)		

The peak latency was not different between the hemispheres for P50m (F_1,10_ = 0.025, p = 0.88), N100m (F_1,10_ = 0.65, p = 0.44), and P200m (F_1,10_ = 0.68, p = 0.43), or among the Prepulse conditions for P50m (F_5,50_ = 1.32, p = 0.27), N100m (F_5,50_ = 0.12, p = 0.99), and P200m (F_5,50_ = 1.30, p = 0.28). Mean peak latencies for all conditions are listed in Table 4.

**Table 4:** Latency in Experiment 2.

Test	P50		N100		P200	
	Left	Right	Left	Right	Left	Right
Control	63.9(11.2)	67.6(6.25)	110(5.82)	108(8.22)	187(22.4)	187(19.6)
+3dB	64.6(10.9)	66.6(10.9)	110(10.4)	114(7.76)	198(17.5)	182(24.9)
+6dB	63.9(10.1)	66.4(10.8)	107(11.5)	113(12.2)	195(26.6)	185(14.7)
+9dB	63.3(10.1)	62.3(9.90)	111(18.7)	109(8.72)	199(18.1)	190(23.2)
+12dB	63.9(10.8)	62.8(7.32)	108(11.0)	114(16.3)	193(25.9)	185(12.2)
+15dB	66.4(11.5)	67.9(11.2)	108(17.9)	114(10.1)	179(15.2)	181(20.7)
Pre						
+3dB	86.1(14.9)	78.6(12.2)	135(11.5)	128(19.6)	224(11.4)	218(18.3)
+6dB	76.8(9.00)	69.1(8.17)	124(9.91)	125(10.9)	215(23.7)	199(13.6)
+9dB	70.5(8.09)	66.0(7.41)	111(9.01)	115(7.67)	190(19.7)	195(22.0)
+12dB	68.3(7.05)	65.0(7.67)	112(10.5)	108(4.62)	191(19.7)	192(13.5)
+15dB	64.5(10.0)	64.3(11.9)	108(8.77)	117(14.5)	188(11.3)	187(18.6)

### Experiment 3

**Fig 6:**
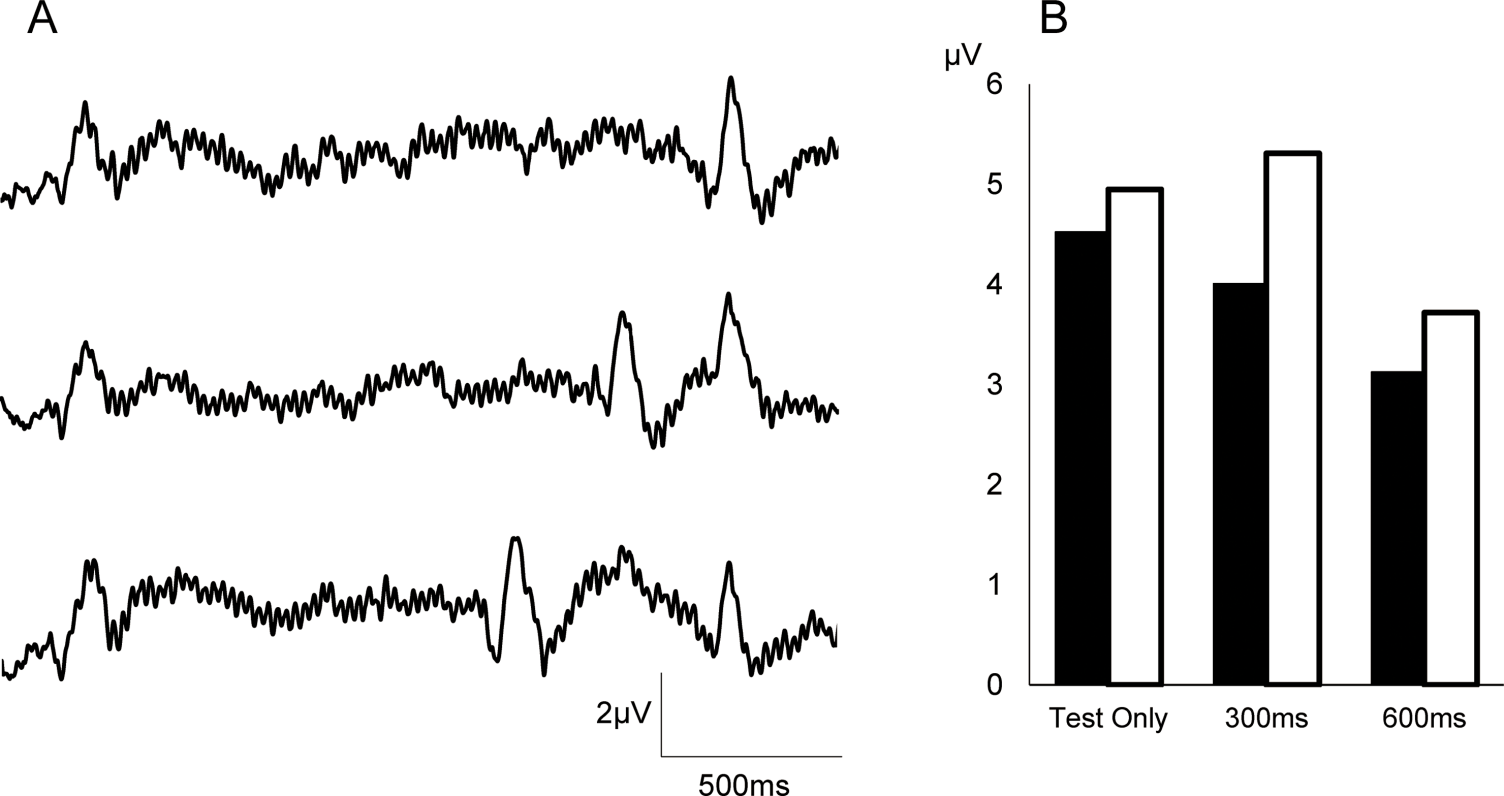
Recordings obtained with EEG. A. Evoked potentials were recorded using Fz referred to linked mastoids. B. Mean amplitude for each condition. Black and white bars show P50-N100 and N100-P200 amplitude, respectively.

Grand-averaged waveforms are shown in Fig 6. The peak amplitude and %inhibition for each condition are listed in Table 5. Both P50-N100 and N100-P200 components were suppressed in a manner similar to the results noted in the MEG experiments. Results of one-way ANOVA showed that Prepulse was a significant factor for determining the amplitude of P50-N100 (F_2,20_ = 5.76, p = 0.011) and N100-P200 (F_2,20_ = 8.63, p = 0.002).

**Table 5:** Rates of amplitude, latency, and inhibition in Experiment 3.

Test	Amplitude		Latency		Test/ Test alone PPI%	
	P50/N100	N100/P200	P50	N100	P50/N100	N100/P200
Control	4.60(1.44)	5.06(2.74)	59.2(11.5)	111(9.10)		
300	4.19(2.49)	5.65(3.18)	58.6(7.60)	105(11.0)	12.1(27.5)	-14.0(19.5)
600	3.10(0.895)	4.02(2.22)	61.0(7.06)	108(10.3)	30.4(15.8)	20.0(24.4)
Pre					Test/Pre PPI%	
300	4.17(1.61)	4.17(1.61)	62.4(9.48)	112(15.1)	-6.09(51.8)	-1.12(26.2)
600	4.47(1.56)	4.47(1.56)	62.2(7.37)	117(10.9)	26.7(19.1)	24.2(19.2)

The correlation of %inhibition between Test alone-based and Prepulse-based inhibition values was examined. Fig 3 shows scatter plots of %inhibition for the 2 calculations. There was a significant correlation with both P50-N100 (r^2^ = 0.40, p = 0.028) and N100-P200 (r^2^ = 0.37, p = 0.015).

The peak latency of the test response was not different between the prepulses for both P50 (F_2,20_ = 1.02, p = 0.38) and N100 (F_2,20_ = 3.17, p = 0.64). In contrast, the difference in latency of P200 was significant (F_2,20_ = 5.57, p = 0.012). That is, the latency of the 300-ms condition was significantly longer than that for the Test-alone (p = 0.007) and Test + 600-ms Prepulse (p = 0.047) conditions (Table 5).

## Discussion

### Methodological considerations

#### Stimulus interval and sound pressure

Although several previously reported studies used P50 paired-stimulation paradigms, the stimulation and measuring methods utilized varied. On the other hand, only a few studies have evaluated the available methodologies. To the best of our knowledge, 2 previous studies evaluated the appropriate stimulation paradigm by manipulating the CTI [28, 29]. Nagatomo et al. (1989) used CTIs of 75, 150, and 500 ms and found that P50 suppression was greater with a longer CTI. More importantly, they found that suppression was clearly weaker for patients with schizophrenia as compared to normal controls at longer CTIs. Adler et al. (1982) examined 500-, 1000-, and 2000-ms CTIs and found that a CTI of 500 ms for the normal controls had a greater than 90% mean decrement in response, while with that of 2000 ms, the inhibitory effects were diminished. These findings appear to be the basis of the 500-ms CTI employed in the paired-stimulation paradigm. However, systematic exploration at around 500 ms has not been done, likely due to the extended experiment time needed, as such a study is quite exhaustive. In the present study, we examined CTIs of 300–800 ms in detail and our results showed significant differences in regard to the degree of suppression, with the greatest amount of suppression seen with a CTI of 600–700 ms.

In our previous study, we used change-related auditory responses and reported that the %inhibition value was about 10% when a weak change stimulus (5 dB increase from the background) was used as a prepulse, suggesting a higher threshold for long-latency as compared to short-latency suppression [23]. As confirmation of this finding, the present results obtained in Experiment 2 showed that an increase of more than 12 dB from the baseline was necessary to obtain a %inhibition greater than 25%. Therefore, a prepulse identical to the Test condition appeared to be suitable for clinical use. To our knowledge, no previous study has examined the effects of the sound pressure of the conditioning stimulus.

#### Trials comprised of testing alone are not necessary

It is known that the amplitude of a change-related response is dependent on the duration of the steady state preceding the onset of change [15, 17, 18, 27], which reflects the behavior of echoic memory involved in generating a change-related response [27]. In previous studies, when the duration of the steady state prior to onset of change varied, the change-related response amplitude was steeply increased from 25–500 ms, whereas that increment was modest at longer durations because of the non-linear temporal nature of echoic memory [17, 27]. We considered this to be the main reason to explain why the Prepulse-evoked responses did not differ in amplitude among the conditions used in the present study. In Experiment 1, we found that the %inhibition values were significantly correlated when calculated against the Prepulse-evoked response and Test alone response. Taken together, these findings indicate that a Prepulse-evoked response may be an alternative to a Test-alone response in the present paradigm, thus shortening the inspection time. If only a Test + 600-ms Prepulse stimulus is necessary, then 8 minutes would be required to complete 200 trials.

#### P50, N100, and P200 components

Standard paired-stimulation suppression paradigms were used to evaluate the P50 component. Although the P50 suppression paradigm is widely used and its clinical importance is evident, as shown in many studies that confirmed suppression deficiency in patients with schizophrenia [1–6], the poor signal-to-noise ratio of P50 can be problematic and some studies have reported low test-retest reliability in regard to suppression ratio [14]. On the other hand, others have reported significantly higher degrees of test-retest reliability of N100 and P200 suppression in schizophrenic patients [30, 31]. In addition, it was shown that the genetic influences on P50 suppression are modest, while the heritability of N100 and P200 gating is high and significant [32]. A study regarding epilepsy also noted that P200, but not P50 or N100, was a useful index for suppression deficits [33]. Therefore, measurements of long-latency components as well as P50 appear to be valuable. In the present study, the behavior of P50-N100 and N100-P200 were not different in regard to long-latency suppression.

Although the inhibitory effect was greatest for the 600–700 ms CTI conditions, Prepulses under those conditions did not affect the latency of the Test-evoked response. Rather, the P200 latency of the Test response was significantly longer for the Test + 300-ms Prepulse condition. These results suggested that there are some mechanisms, other than the long-latency suppression in question, that affect Test response at shorter CTIs. Although the significant effect on the P200 latency is interesting, further studies are necessary to validate our findings, since P200 measurements show the largest dispersion among the 3 components used [34–36].

We used pure tones instead of clicks in the present study, because results of our preliminary study showed that pure tones can elicit clearer change-related cortical responses as compared to clicks, particularly when using EEG. Change-related cortical responses are elicited by an abrupt change in sound feature, regardless of whether the sound is composed of clicks or pure tones [17]. One merit of clicks is that they are superior to pure tones for eliciting a P50 component [19, 30]. Therefore, if the focus is on the P50 component, use of clicks is preferable.

#### Onset response and change-related response

For the Test response, we adopted a change-evoked response, while standard P50 suppression paradigms use conventional onset responses. This raises the possibility that these two test responses as well as their suppression are controlled by different mechanisms. However, the onset response can be regarded as a type of change-related response [16] and some studies have reported a close relationship between onset and change-related responses [16–21]. Therefore, we considered that the present paired-pulse suppression and P50 suppression reflected similar, if not identical, inhibitory processes. The fact that both test responses showed maximum suppression with a CTI of approximately 500 ms supports this view.

### Long-latency suppression at approximately 600 ms

#### Active inhibitory process

The present results showed that suppression by the Prepulse was significant for 500–800 CTIs, but not for a CIT of 300 or 400, suggesting that the reduction in response magnitude was not due to refractoriness or fatigue of pyramidal cells (PCs), or PC-PC transmission, but rather mainly because of active inhibitory processes. Although local inhibitory circuits have many functions including sharpening of spike timing [37], such a long-latency and high-threshold inhibitory system may appear to function as a gate for excessive excitation of PCs. In fact, gating is one of the roles played by inhibitory circuits [38]. From the view point of cytophysiology, there are more than 10 different interneuron types in the macaque cortex [39], which induce inhibitory postsynaptic potentials (IPSPs) by releasing GABA. Interneurons can be roughly divided into 3 groups [40]. The first is comprised of parvalbumin-expressing cells, classified morphologically as basket and chandelier cells [41, 42], which form inhibitory synapses to the PC body. Another group expresses calretinin and these cells terminate around other interneurons. The third group is somatostatin-positive Martinotti cells [43, 44]. Martinotti cells form inhibitory synapses to PCs and mediate long-range inhibition that rises to surround suppression via GABA-A receptors [22, 45], while they are also known to generate long-latency IPSPs, with that latency dependent on the number or frequency of input spikes [22]. Therefore, it may be likely that the present paired-stimulation suppression reflects IPSPs of Martinotti cells in terms of latency. The results of Experiment 2 also support this notion, as the suppression showed a higher threshold as compared to short-latency suppression [23]. Martinotti cells show a high threshold to induce IPSPs and do not respond with low-frequency inputs [22]. However, there remains the possibility that the present long-latency suppression was mediated by unknown interneurons.

### Importance for clinical testing

The results of Experiment 3 showed that the inhibitory process can be detected using EEG, a technique that is advantageous in terms of cost, convenience, and availability. Furthermore, the P50 suppression paradigm has been employed in several studies and is useful to evaluate inhibitory processes [1–13]. As a rule, electrophysiological measures of such a long-latency event need long inspection time. A previous study attempted to shorten the recording time in the P50 suppression paradigm by changing the trial-trial interval from 10 to 5 seconds and their results indicated that it might be possible [46]. In this regard, the present method, taking around 6 minutes, is relatively short as compared to other electrophysiological methods. Because deficits in the inhibitory system is considered to be involved in pathophysiology of many diseases such as schizophrenia [47, 48] and epilepsy [49, 50], the present method may be useful clinically. However, the limitation of this study includes the wide range of subject’s age (20–53 years old) and small sample size. In order to establish clinical significance of the present paired-pulse suppression, future studies with a larger sample of both controls and patients are necessary.
